# Effect of Oat Hay as a Substitute for Alfalfa Hay on the Gut Microbiome and Metabolites of Yak Calves

**DOI:** 10.3390/ani14223329

**Published:** 2024-11-19

**Authors:** Yingchao Gu, Lele An, Yanan Zhou, Guoliang Xue, Yang Jiao, Deyu Yang, Shujie Liu, Zhanhong Cui

**Affiliations:** 1 Qinghai Academy of Animal Husbandry and Veterinary Sciences, Qinghai University, Xining 810016, China; gyc1325935243@163.com (Y.G.); anlele0608@126.com (L.A.); yanan_zhou0312@163.com (Y.Z.); annxgl12345@163.com (G.X.); jiaoyang202208@126.com (Y.J.); 18697175846@163.com (D.Y.); mkylshj@126.com (S.L.); 2 Ministry of Agriculture and Rural Affairs Key Laboratory of Animal Nutrition and Forage-Feed of Grazing Yak and Tibetan Sheep in Qinghai-Tibetan Plateau, Xining 810016, China; 3 Yak Engineering Technology Research Center of Qinghai Province, Xining 810016, China; 4 Key Laboratory of Plateau Grazing Animal Nutrition and Feed Science of Qinghai Province, Xining 810016, China

**Keywords:** jejunum, colon, microbiological composition, differential metabolites

## Abstract

To evaluate the impact of different roughages on the intestinal microbiota of yak calves, we fed them oat hay in substitution of alfalfa hay, in addition to milk replacer and starter powder. Twenty-one 45-day-old male yak calves were selected and randomly assigned to three groups: the milk replacer + starter + alfalfa hay group (AH), the milk replacer + starter + oat hay group (OH), and the milk replacer + starter + mixed hay group (AO), in which the alfalfa hay and oat hay were administered in a 1:1 ratio. All calves in the three groups were fed the same milk replacer and an equivalent amount of dry matter. The formal experiment commenced after a 21-day pre-test period and lasted for 120 days. Following the experiment, the contents of the jejunum and colon were collected to investigate the intestinal microbiota and metabolites using 16S rRNA sequencing and LC-MS metabolomics. The result showed that the AO group had greater final body weights overall than the AH group and OH group (*p* < 0.05). The AH group and OH group had considerably greater feed-to-gain ratios than the AO group (*p* < 0.05). At the phylum level, the OH group exhibited an increased relative abundance of *Bacteroidota* and *Spirochaetota* in the jejunum (*p* < 0.05). The relative abundance of *Actinobacteriota* in the colon was increased in the AO group (*p* < 0.05). At the genus level, the AO group exhibited a decreased abundance of *Clostridium sensu_stricto_1* (*p* < 0.05), and the OH group showed an increased abundance *UCG-005* and *Alistipes* in the jejunum. There were many differential metabolites in the OH group and AO group compared to the AH group, and the different metabolites of the OH group were associated with the metabolic pathways of the nervous system, sensory system, amino acid metabolism, and lipid metabolism in the jejunum and with lipid metabolism, amino acid metabolism, and the nervous system in the colon. In the AO group, these metabolites were associated with the digestive system and the translation and metabolism of cofactors in the jejunum and with the metabolism of cofactors and vitamins in the colon. In summary, it is feasible to replace alfalfa hay with oat hay based on milk replacer and starter. The combination of the two forages enhanced nutrient absorption, improved immune function, maintained the internal homeostasis of yak calves, and was more beneficial to their growth and development.

## Simple Summary

To evaluate the effects of different roughages on the intestinal microbiota of yak calves, oat hay was partially substituted for alfalfa hay and fed to yak calves along with milk replacer and starter. The results of the study showed that yak calves fed a 1:1 mixture of alfalfa hay and oat hay attained higher final body weights but had lower feed conversion ratios. Replacing alfalfa grass with oat grass not only changed the composition of gut microorganisms but also affected metabolite profiles. In addition, the combination of these two hay species improved nutrient absorption and immune function in yak calves.

## 1. Introduction

The yak (Bos grunniens) is an ancient and unique type of grazing ruminant on the Qinghai–Tibetan Plateau (QTP) [1]. In the QTP environment, it fills a crucial ecological niche and maintains a semi-domesticated condition by herding on the plateau meadow and through natural breeding [2]. Female yaks typically give birth once every two years. According to traditional breeding practices, yak calves are typically nursed by cows until they are 1.5 to 2 years old before being weaned either naturally or artificially [1]. Due to nutritional deficiencies and hard climatic circumstances, calves managed in this manner grow slowly and exhibit significant death rates [3]. The early feeding management model is crucial for the growth of calves and the later production capacity, which can have long-term impacts, according to several studies [4]. Our team adopted milk replacer, concentrate feed, and alfalfa hay for yak calves raised by stall-feeding in the early stage with remarkable results in terms of the growth performance of yak calves and the nutritional structure of the diet [5,6,7].

Although alfalfa hay is frequently utilized in large quantities for dairy industry breeding, its low yield and substandard quality cannot keep up with the demand generated by the accelerated growth of animal husbandry in China, resulting in a heavy reliance on alfalfa hay imports [8]. To mitigate food competition with dairy cows and reduce feeding costs, we utilize regional roughage resources in Qinghai Province, specifically oat hay, a grass rich in fermentable fiber and soluble carbohydrates, to partially or completely substitute alfalfa hay in the breeding of yak calves. This study aims to investigate the effects of various roughages on the intestinal microbiota of yak calves and to provide guidance for selecting suitable roughages for the early rearing of yak calves.

Healthy gastrointestinal development serves as the foundation for nutrient deposition and animal growth. The intestinal tract is an important part of feed digestion and nutrient absorption [9,10]. Furthermore, the gut is inhabited by diverse bacteria, rendering it a complex endocrine organ. The trillions of gut microbiota cells regulate health and bridge the gap between the host’s dietary intake and physiological function [11]. Notably, gut microbes are of utmost importance during the early stages of ruminant life, even more so than in later stages [12]. Recent studies have revealed that calves possess a limited number of metabolically active microorganisms in their gut from the fetal period onward [13]. The intestinal microbiota of calves undergo a dynamic and gradual transition, evolving from colonization to stabilization. This transition is closely linked to the development of the gastrointestinal tract (GIT), particularly during the weaning period. The gastrointestinal microbial composition of young ruminants is significantly and persistently influenced by alterations in pre-weaning diet and feeding patterns [14,15].

The morphological structure of each intestinal segment in calves varies, resulting in distinct microbiota populations within different regions of the gut. Numerous researchers have observed significant and enduring alterations in intestinal microorganisms that arise from changes in the diet and feeding practices of pre-weaned calves. The administration of different nutrients to calves influences the microflora structure by modulating the colonization and establishment of intestinal microbes [16]. Nevertheless, there remains a gap in our understanding of how the gut microbiome of yak calves evolves and adapts, particularly in response to the introduction of roughage.

In this paper, yak calves were fed with different proportions of oat hay instead of alfalfa hay, and 16S rRNA sequencing and LC-MS metabolomics were used to study the intestinal microbiota and metabolites in order to determine the effect of different feeds on the calf’s intestinal tract.

## 2. Materials and Methods

### 2.1. Experimental Animals and Group Design

This animal experiment and its related operations were approved by the Laboratory Animal Management Committee of the Qinghai Academy of Animal Husbandry and Veterinary Sciences (2022-QHMKY-015). In this experiment, 21 healthy male yak calves aged 45 days were used as test animals. The animals were ranked based on their initial weights and allocated to three blocks of seven animals each. Subsequently, the calves were randomly assigned to three treatment groups: the milk replacer, starter, and alfalfa hay group (AH); milk replacer, starter, and oat hay group (OH); and milk replacer, starter, alfalfa hay, and oat hay group (AO) (alfalfa–oat ratio 1:1). The calves in all three groups received an equal quantity of dry matter and milk replacer. The formal trial phase, which commenced 21 days after the pre-feeding period, lasted for 120 days. The experiment was conducted in the pastures of the Plateau Ecological Animal Husbandry Science and Technology Demonstration Park of Haibei Tibetan Autonomous Prefecture in Qinghai Province (36°92′ N, 100°96′ E), located at an elevation of 3010 m and with an average yearly temperature of 1.5 °C. Sample measurements were taken at the Key Laboratory of Plateau Grazing Animal Nutrition and Feed Science at Qinghai University, as well as at the Qinghai Academy of Animal Husbandry and Veterinary Sciences.

### 2.2. Breeding Management and Test Diet

After a 21-day pre-testing period, the three groups of yak calves were acclimatized to their respective diets. Subsequently, the official 120-day test period commenced. During the entire experiment, each calf was housed in a separate enclosure with unrestricted access to food and water. The pens were cleaned and sanitized weekly. All three groups received the same milk replacer and starter, and had ad libitum access to alfalfa and oat hay, as per their experimental groupings. The quantity of solid dry matter provided was uniform across all groups. Each yak was fed two times a day with 0.48 kg of milk replacer, which was increased by 15 g every seven days. Each animal also consumed 140 g of roughage per day, with a 70 g increase every seven days. The starter feed was 70 g per day, and it increased by 30 g every seven days. During feeding, the concentrate and roughage were fed separately, with the concentrate being fed first and then the roughage. The milk replacer was prepared in bottles filled with boiled and cooled water at approximately 42 °C, which the calves then suckled from. The oat hay and alfalfa hay were sourced from Haiyan County in Qinghai Province and Zhangye City in Gansu Province, respectively. The milk replacer and starter feed were purchased from the Beijing Precision Animal Nutrition Research Center. The hay was crushed to a length of 3 to 5 cm. Table 1 presents the nutrient contents of the diets.

### 2.3. Sample Collection and Processing

The feeding trial was terminated when the total dry matter intake of roughage and concentrate reached 1 kg per yak calf (calves were 186 days old at this time), followed by slaughtering and sampling. After the test and 12 h of fasting, five calves per group were randomly selected for slaughter, and the abdominal cavity was opened for dissection. To separate the intestinal tissue, the jejunum and colon were folded in half, and 8–10 g of contents were collected from the middle portion of the intestinal segments, which were taken out and divided into four 5 mL sterilized centrifuge tubes. These tubes were then immediately flash frozen in liquid nitrogen and moved to a −80 °C refrigerator for the analysis of metabolites and the composition of the intestinal microbiota.

### 2.4. DNA Extraction, 16S rDNA Gene Amplification, High-Throughput Sequencing, and Bioinformatics Analysis

The whole-genome DNA in the samples was extracted using the CTAB/SDS technique. Using 1% agarose gels, DNA concentration and purity were examined. DNA was diluted to a concentration of 1 ng/L using sterile water.

Using the particular primer sets 515F (50-GTGCCAGCMGCCGCGG-30) and 806R (50-GGACTACNNGGGTATCTAAT-30) and barcodes, the 16S rRNA genes of the 16S V3–V4 regions were amplified. The cycling conditions were a first denaturation step at 98 °C for 1 min; followed by 30 cycles at 98 °C (10 s), 50 °C (30 s), and 72 °C (30 s); and a final 5 min extension at 72 °C. All PCR samples contained 15 L of Phusion^®^ High-Fidelity PCR Master Mix with GC Buffer (New England Biolabs, Inc., Ipswich, MA, USA), 0.2 M of each primer, and 10ng target DNA.

To remove the non-specific products, all of the PCR products were examined and purified using 2% agarose gel electrophoresis and 1X loading buffer (containing SYB green). Afterward, the combined PCR products were purified using a Qiagen Gel Extraction Kit (Qiagen, Hilden, Germany). Using the NEBNext^®^ UltraTM IIDNA Library Prep Kit (Cat No. E7645) (New England Biolabs, Inc.), sequencing libraries were created following the manufacturer’s instructions. A Qubit@ 2.0 Fluorometer from Thermo Scientific and an Agilent Bioanalyzer 2100 system (Agilent Technologies, Santa Clara, CA, USA) were used to assess the library’s quality. Finally, 250 bp paired-end reads from the sequencing of the library on the Illumina NovaSeq platform were produced.

The following procedures were used to obtain more dependable and high-quality sequencing results: To produce high-quality clean tags, fastp (Version 0.20.0) software was used to perform quality filtering on the raw tags. Using Vsearch (Version 2.15.0), chimeric sequences were found within the clean tags by comparing them to the reference database (Silva version 138.1), and the chimeric sequences were then eliminated to produce the effective tags. The acquired sequences were then grouped into OTUs (operational taxonomic units) using Uparse software (Uparse version 7.0.1001) at a sequence similarity of above 97%. A Venn diagram was created using R (v3.0.3) to graphically represent the amount of shared and unique OTUs among various groups. Using QIIME 2 software, four indicators—Chao1, Shannon, Simpson, and Good’s coverage—were computed to assess alpha diversity; they represented the species richness and species diversity of samples to determine which species were substantially different across sets of samples in terms of microbial community abundance at each taxonomic level (phylum, genus). After testing the normality and homoscedasticity of the residuals, *t*-test analyses were performed using the R program (version 3.5.3).

### 2.5. Untargeted Metabolomics Data Processing and Metabolite Identification

The intestinal contents (100 μL) were resuspended in Eppendorf tubes by well vortexing with prechilled 80% methanol. The samples were then placed on ice and incubated for 5 min, followed by centrifugation at 15,000× *g* for 20 min at 4 °C. A portion of the supernatant obtained by centrifugation was diluted with LC-MS-grade water to a final concentration of 53% methanol. Subsequently, the sample was transferred to new Eppendorf tube and centrifuged under the same conditions. Finally, the supernatant was injected into the LC-MS/MS system for analysis.

UHPLC-MS/MS studies were carried out by Novogene Co., Ltd. (Beijing, China) using a Vanquish UHPLC system (Thermo Fisher, Bremen, Germany) paired with an Orbitrap Q ExactiveTM HF mass spectrometer (Thermo Fisher, Bremen, Germany). Peak alignment, peak selection, and quantification for each metabolite were carried out using Compound Discoverer 3.1 (CD3.1, Thermo Fisher) to handle the raw data files produced by UHPLC-MS/MS.

Principal components analysis (PCA) and partial least squares discriminant analysis (PLS-DA) were carried out using metaX (a versatile and all-inclusive program for processing metabolomics data), and these metabolites were identified using the KEGG database (https://www.genome.jp/kegg/pathway.html accessed on 10 February 2018). Statistical significance (*p*-value) was calculated using univariate analysis (*t*-test). The metabolites were regarded as differential metabolites if they exhibited VIP > 1 and *p* < 0.05, a fold change of 2, or an FC of 0.5. The metabolites of interest were filtered using volcano plots based on the log2 (fold change) and log10 (*p*-value) of the metabolites using ggplot2 in R. Enrichment analysis of metabolic pathways for the differential metabolites was carried out; when the ratio was satisfied by x/n > y/N, the enrichment of the metabolic pathways was confirmed; when the *p*-value for the enrichment of the metabolic pathways was 0.05, the enrichment of the metabolic pathways was deemed statistically significant.

### 2.6. Statistical Analysis

In this study, the Spearman statistical approach was used to determine the correlation coefficient rho between the relative abundance of different metabolites and the overall abundance of different bacteria (rho = 0, *p* = 0.05). One-way ANOVA using SPSS 22.0 software (SPSS, Inc., Chicago, IL, USA) was used for growth performance index analysis with replicates as experimental units, and values *p* < 0.05 were considered statistically significant. All data are expressed as means with the SEM.

## 3. Results

### 3.1. Effects of Oat Hay as a Substitute for Alfalfa Hay on the Growth Performance of Yak Calves

The initial body weight, final body weight, average daily weight gain, feed-to-gain ratios ratio, and dry matter ingested are presented in Table 2. The calves initially selected for the study exhibited similar weights. However, the calves in the AO group had significantly higher final body weights overall compared to those in the AH and OH groups (*p* < 0.05). Additionally, the AH and OH groups had considerably higher feed-to-gain ratios than the AO group (*p* < 0.05), whereas the AO group had the lowest ratio.

### 3.2. Effect of Oat Hay as a Substitute for Alfalfa Hay on the Intestinal Microbial Diversity of Yak Calves

**Figure 1:**
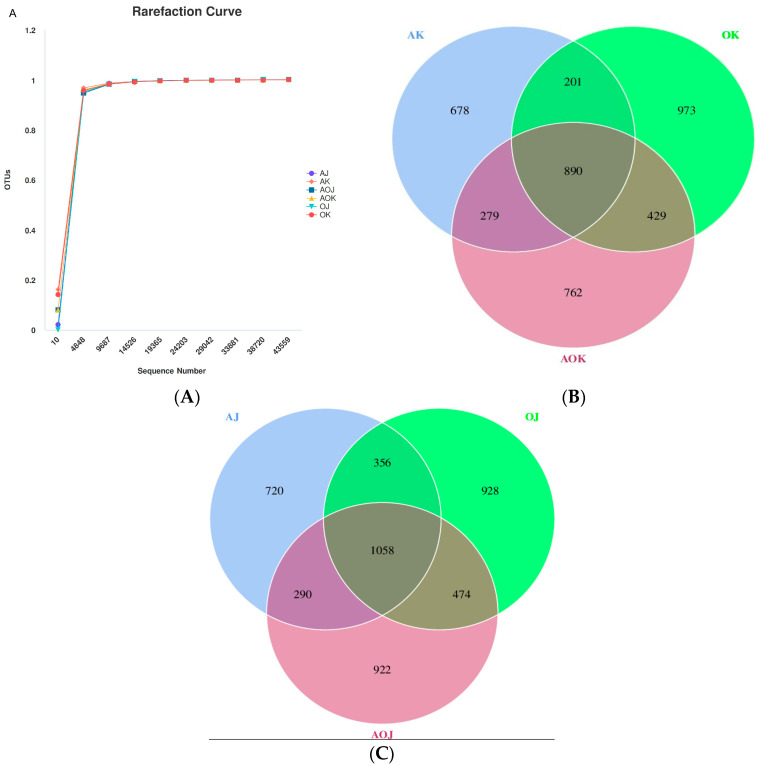
(**A**) Rarefaction curves of all groups. (**B**) The Venn diagram shows the bacterial composition of OTUs in the jejunum. (**C**) The Venn diagram shows the bacterial composition of OTUs in the colon. AK, OK, and AOK represent the jejunum of the AH, OH, and AO groups, respectively; while AJ, OJ, and AOJ represent the colon of the AH, OH, and AO groups, respectively.

The amplicon sequencing technique was employed to evaluate the extent of diversification in the intestinal microbiome’s functional potential when animals were fed diets containing various roughage sources. Following rigorous sequence quality filtering, a total of 1,801,910 effective tags (clean reads) were obtained from 30 intestinal content samples. The Good’s coverage exceeded 99% (Figure 1A), indicating that the diversity coverage of the samples reached a high level and that the sequencing depth was nearly saturated. This ensures that the sequencing results accurately represent the microbial composition of the samples.

In the jejunum, a total of 4219 OTUs were identified across all experimental groups, with 890 OTUs common to all samples. Notably, 678 OTUs were unique to group AH, 973 were exclusive to group OH, and 762 were specific to group AO (Figure 1B). This variation suggests that different dietary roughage sources influence the microbial composition in the jejunum. Turning to the colon, a broader analysis revealed a total of 4337 OTUs across the various feeding groups. Remarkably, 4087 of these OTUs were present in all samples (Figure 1C), indicating a core microbiome shared among the groups. However, there were also specific OTUs that were unique to each group: 91 OTUs were exclusive to group AO, 26 were specific to group OH, and 11 were unique to group AH. These findings highlight the distinct effects of different roughage sources on the colonic microbiome composition.

There were variations in the number and diversity of microorganisms in various groups, which we evaluated using a range of alpha diversity indices. The results indicated that Chao1 indices, representative of the richness of microbiome communities, increased sequentially from the AH group to the OH group and further to the AO group in the jejunum. When compared to the AH group, the AO group had a significantly higher score (*p* < 0.05), demonstrating that substituting oat hay for alfalfa hay enhanced the number of microbial species in the jejunum, with the optimal outcome achieved when half of the oat hay was used as a replacement. Conversely, the AH group significantly underperformed compared to the other two groups (*p* < 0.05) in terms of colon Chao1 and Shannon indexes of the colon, among other metrics (Table 3). Additionally, the Simpson index and Shannon index further indicated both richness and evenness. This finding implies that, similarly to the microorganisms in the jejunum, the microbial population in the colon also increased when alfalfa hay was gradually replaced with oat hay.

**Figure 2:**
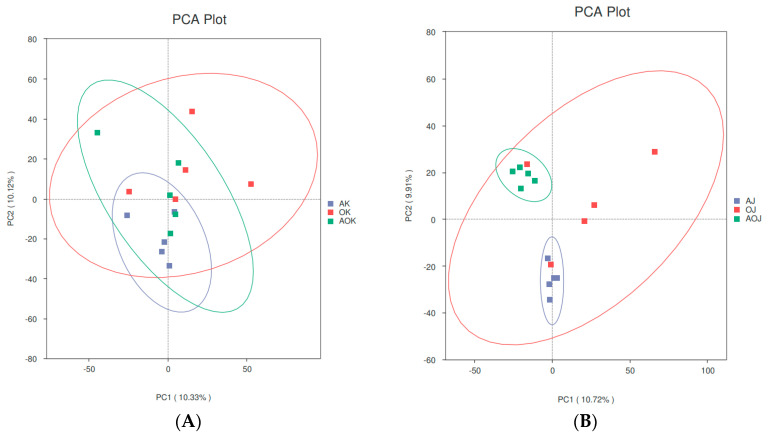
Beta diversity (**A**) PCA scatter plot of jejunum; (**B**) PCA scatter plot of colon.

Beta diversity was calculated to assess the differences between the three groups of gut microbial communities. The organization and content of the colon microbiome in calves was altered when oat feed was substituted for alfalfa hay, as can be seen in the scatterplot using PCA. The difference between the AH group and the AO group, in particular, was significantly greater (Figure 2B). Additionally, the scattered points in the intestinal microbiome of group OH were more dispersed, indicating high variability and potential instability in the microbiome of yak calves.

**Figure 3:**
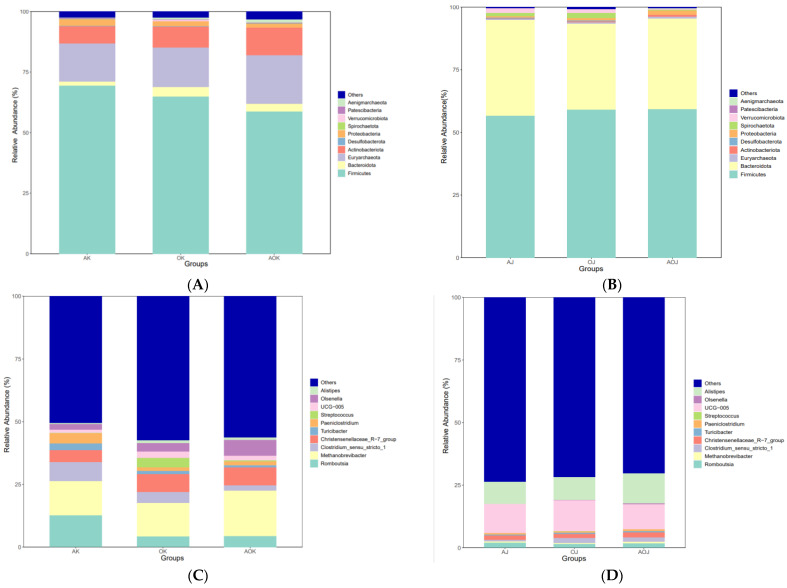
(**A**) Top 10 species in abundance at the phylum level in the jejunum. (**B**) Top 10 species in abundance at the phylum level in the colon. (**C**) Top 10 species in abundance at the genus level in the jejunum. (**D**) Top 10 species in abundance at the genus level in the colon.

We evaluated the relative abundance of the dominant bacteria at both the phylum and genus taxonomical levels in each sample. Across all groups, Firmicutes emerged as the most abundant phylum in both the jejunum and colon, accounting for over 56.63% of the total sequences. In the jejunum, Euryarchaeota followed as the second most dominant phylum, with the AH group showing 15.71%, the OH group 16.22%, and the AO group 20.16%. In contrast, in the colon, Bacteroidota took the second spot, with abundances of 38.24% in the AH group, 34.42% in the OH group, and 36.27% in the AO group (Figure 3A,B).

At the genus level, Romboutsia and Methanobrevibacter were significantly enriched in the jejunum. Specifically, Romboutsia abundances were 12.72% in the AH group, 4.18% in the OH group, and 4.41% in the AO group. Similarly, Methanobrevibacter abundances were 13.67% in the AH group, 13.48% in the OH group, and 18.18% in the AO group. In the colon, UCG-005 and Alistipes were the notable genera. UCG-005 abundances were 11.55% in the AH group, 12.47% in the OH group, and 10.02% in the AO group. Meanwhile, Alistipes abundances were 8.93% in the AH group, 9.00% in the OH group, and 12.07% in the AO group (Figure 3C,D).

**Figure 4:**
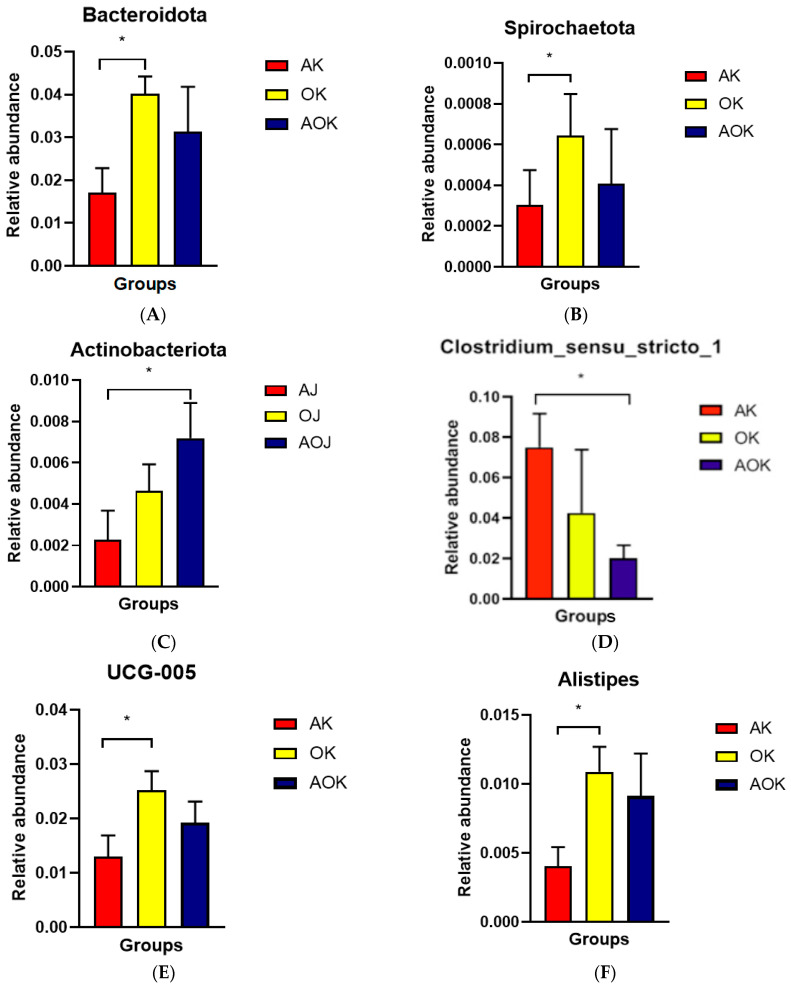
(**A**,**B**) Bacteria with differential relative abundance at the phylum level in the jejunum. (**C**) Bacteria with differential relative abundance at the phylum level in the colon. (**D**–**F**) Bacteria with differential relative abundance at the genus level in the jejunum. “*” means *p* < 0.05.

Subsequently, *t*-tests were performed to compare the OH group with the AH group and the AO group with the AH group, aiming to precisely delineate the disparities in microbial composition among distinct groups. Within the jejunum, Bacteroidota and Spirochaetota exhibited a notably higher prevalence in the OH group compared to that in the AH group (*p* < 0.01) (Figure 4A,B). Conversely, in the colon, the AO group demonstrated a significantly increased abundance of Actinobacteria relative to that in the AH group (*p* < 0.05) (Figure 4C). At the genus level, within the jejunum, UCG-005 and Alistipes were significantly enriched in the AO group compared to the OH group (*p* < 0.05), whereas Clostridium_sensu_stricto_1 displayed a significantly reduced abundance in the AO group in comparison to the AH group (*p* < 0.05) (Figure 4D–F).

### 3.3. Effect of Oat Hay as a Substitute for Alfalfa Hay on the Gut Metabolic Profiles of Yak Calves

**Figure 5:**
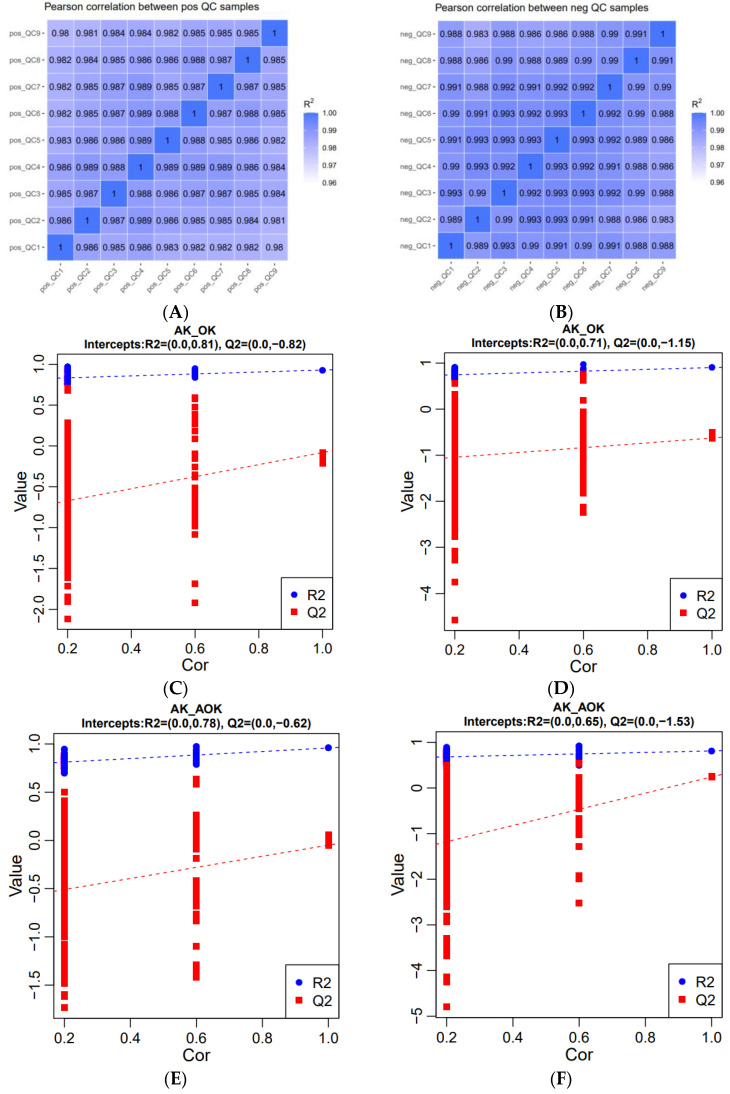

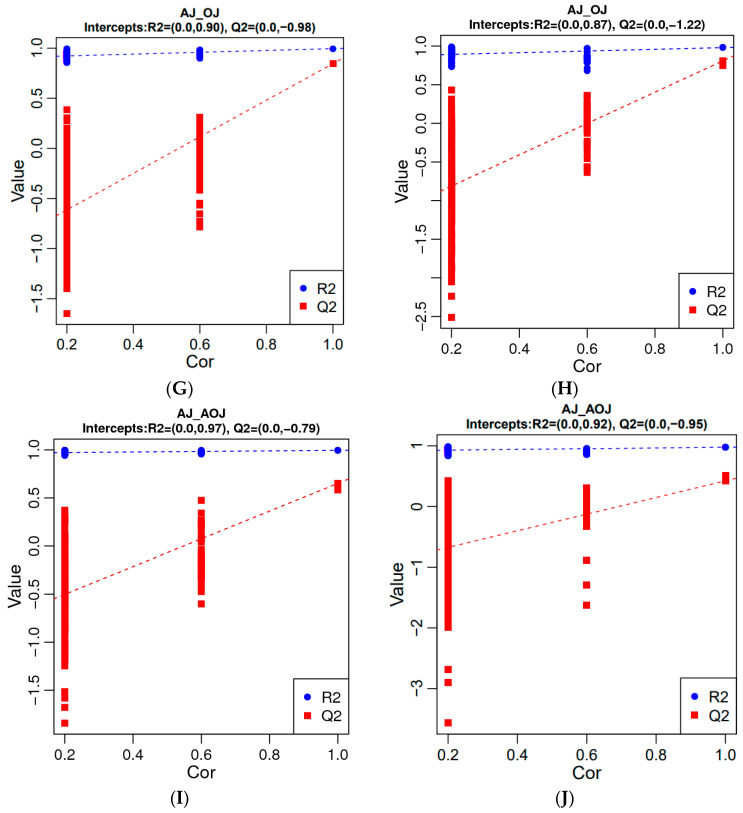
(**A**) Pearson correlation between QC samples in positive ion mode. (**B**) Pearson correlation between QC samples in negative ion mode. (**C**,**D**) PLS-DA between different groups in the jejunum in positive ion mode. (**E**,**F**) PLS-DA between different groups in the jejunum in negative ion mode. (**G**,**H**) PLS-DA between different groups in the colon in positive ion mode. (**I**,**J**) PLS-DA between different groups in the colon in negative ion mode.

Equal amounts of experimental and quality control (QC) samples were combined to test the system’s stability. The closer the R^2^ value is to 1, indicating a stronger correlation, the more stable the entire assay procedure becomes, resulting in higher-quality outcomes. Figure 5A,B demonstrates that the measurement data quality is quite good. Upon positive- and negative-mode ionization, projection to latent structures discriminant analysis (PLS-DA) was used to analyze the QC samples, aiming to describe the general metabolomic variations across various groups. According to the PLS-DA permutation test, the original points on the right exhibited higher values than the R2 and Q2 values on the left. Additionally, the Q2 regression curve has a negative intercept, which demonstrates the reliability and validity of the PLS-DA model (Figure 5C–J).

The differential metabolites were screened with reference to the three parameters of variable influence on projection (VIP), fold change (FC), and *p*-value calculated from a *t*-test of the first principal component of the PLS-DA model, with a threshold of VIP > 1.0, FC > 1.2 or FC < 0.883, and *p* < 0.05.

**Figure 6:**
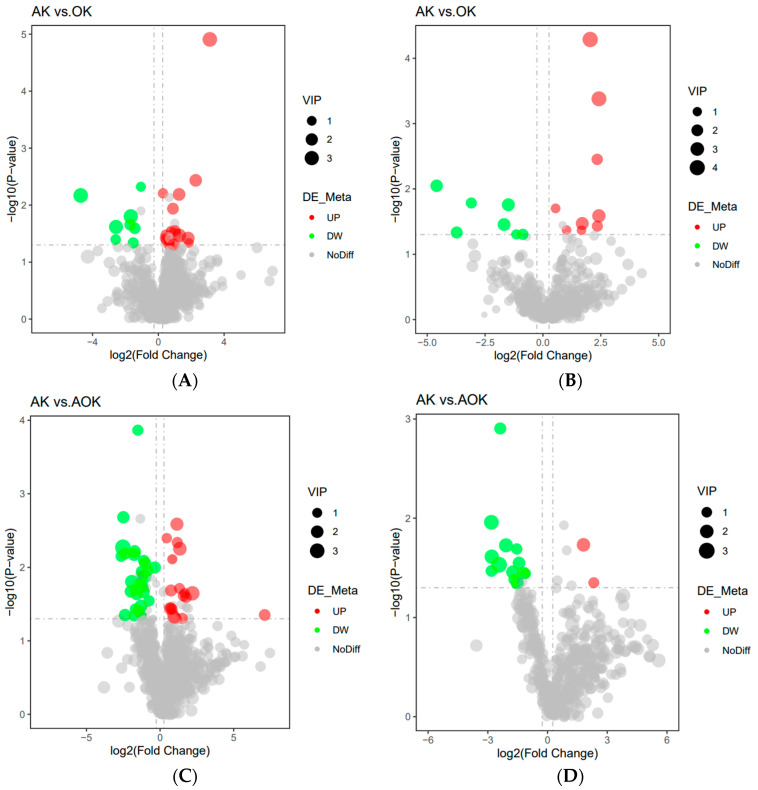
(**A**) Volcano plot of differential metabolites in the jejunum in positive ion mode. (**B**) Volcano plot of differential metabolites in the jejunum in negative ion mode. (**C**) Volcano plot of differential metabolites in the jejunum in positive ion mode. (**D**) Volcano plot of differential metabolites in the jejunum in negative ion mode.

Analysis of the jejunal positive ion pattern showed a total of 25 different metabolites in the OH and AH groups, with 8 metabolites upregulated and 17 metabolites downregulated in the AO group (Figure 6A). A total of 16 distinct metabolites were found to vary between the AH and OH groups in negative ion mode, with 7 metabolites considerably upregulated and 9 significantly downregulated in OH group (Figure 6B). A total of 48 differential metabolites were found in the AH and AO groups, with 29 significantly upregulated and 19 significantly downregulated in the AO group (Figure 6C) and 16 differential metabolites in negative ion mode, with 14 significantly upregulated metabolites and 2 significantly downregulated metabolites in the AO group (Figure 6D).

**Figure 7:**
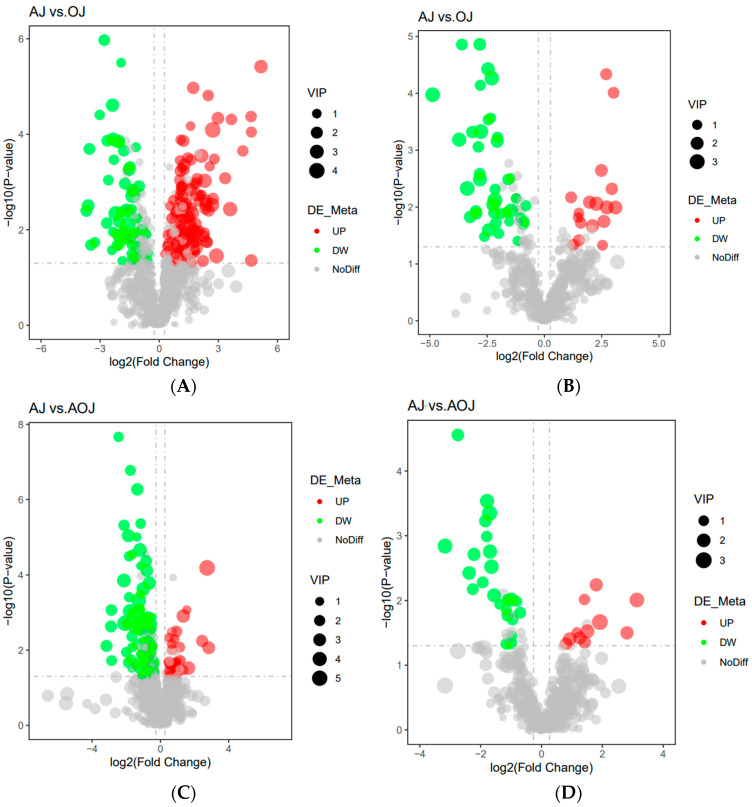
(**A**) Volcano plot of differential metabolites in the colon in positive ion mode. (**B**) Volcano plot of differential metabolites in the colon in negative ion mode. (**C**) Volcano plot of differential metabolites in the colon in positive ion mode. (**D**) Volcano plot of differential metabolites in the colon in negative ion mode.

Analysis of the jejunal positive ion pattern revealed a total of 25 distinct metabolites in the OH and AH groups. Specifically, 8 metabolites were upregulated in the AO group, while 17 metabolites were downregulated (Figure 7A). In contrast, in negative ion mode, 16 metabolites exhibited variation between the AH and OH groups. Notably, seven metabolites were significantly upregulated in the OH group, and nine metabolites were downregulated (Figure 7B). Additionally, a comprehensive comparison between the AH and AO groups yielded a total of 48 differential metabolites. Among these, 29 metabolites were significantly upregulated in the AO group, while 19 were downregulated (Figure 7C). Similarly, in negative ion mode, 16 differential metabolites were observed, with 14 metabolites significantly upregulated and 2 metabolites downregulated in the AO group (Figure 7D).

KEGG pathway enrichment analysis was employed to identify the most significant signal transduction and biological metabolic pathways associated with differential metabolites. The primary foci of the enriched KEGG pathways at level 1 for the jejunum included organismal systems, cellular processes, genetic information processing, and metabolic pathways. Furthermore, the key components of KEGG pathways at level 2 encompassed the neurological system, sensory system, and digestive system, as well as processes such as translation, metabolism of cofactors and vitamins, amino acid metabolism, and lipid metabolism.

Table 4 displays the differential metabolites related to the KEGG pathways in which jejunum metabolites are considerably enriched. The KEGG pathways “inflammatory mediator regulation of TRP channels pathway”, “gap junction”, “synaptic vesicle cycle pathway”, and “taste transduction pathway” were enriched for differential metabolites of serotonin in the OH group and AH group when measured in positive ion mode. Notably, the OH group exhibited significantly lower levels of these metabolites. Conversely, under negative ion mode, no distinct metabolites were observed between the OH group and AH group. Alternatively, in the differential metabolites between the AO group and AH group, 3-methyl-2-oxobutanoic acid panthenol was considerably abundant in the KEGG pathways “valine, leucine, and isoleucine degradation pathway” and “pantothenate and CoA biosynthesis pathway” in the AO group. Additionally, among differential metabolites, the KEGG pathway “pantothenate and CoA biosynthesis” showed a substantial enrichment of D-panthenol. Differential L-palmitoylcarnitine metabolites were enriched in the positive ion mode in the KEGG pathways “fatty acid metabolism pathway” and “fatty acid degradation pathway”. In the AO group, there was a considerable upregulation of all the differential metabolites indicated above. Furthermore, the AO group and AH group exhibited distinct KEGG pathway enrichment under negative ion conditions. The findings indicate that the “aminoacyl-tRNA biosynthesis pathway”, “cysteine and methionine metabolism pathway”, “protein digestion and absorption pathway”, and “biosynthesis of amino acids pathway” were significantly enriched for methionine and L-serine. Additionally, KEGG pathways including “glycine, serine, and threonine metabolism”, “sphingolipid metabolism”, and “sphingolipid signaling pathway” exhibited substantial enrichment for L-serine. Notably, several distinct metabolites were significantly elevated in the AO group.

The enriched KEGG pathway level 1 for the colon was focused on metabolism and organismal systems, whereas the enriched KEGG pathway level 2 was mostly composed of pathways for the metabolism of lipids, amino acids, cofactors, and vitamins, as well as the nervous system.

Table 5 presents the KEGG pathways for which colon metabolites exhibited significant enrichment, along with the differential metabolites associated with these pathways. In positive ion mode, the OH group and AH group exhibited differential metabolites, including adrenic acid, eicosapentaenoic acid, nervonic acid, and docosapentaenoic acid, which are enriched in the KEGG pathway “biosynthesis of unsaturated fatty acids”. Additionally, differential metabolites, including *N*6,*N*6,*N*6-trimethyl-l-lysine, 5-hydroxy-l-lysine, l-hydroxylysine, and l-pipecolate, were enriched in the “lysine degradation pathway”. Notably, these differential metabolites were significantly downregulated in the OH group. In negative ion mode, differential metabolites 4-hydroxybenzoic acid and 7,8-dihydrofolate were enriched in the KEGG pathway “folate biosynthesis”, while 3-methoxy-4-hydroxyphenylacetate was enriched in the KEGG pathway “dopaminergic synapse”. Furthermore, the differential metabolites 4-hydroxybenzoic acid and 3-methoxy-4-hydroxyphenylacetate were downregulated, whereas 7,8-dihydrofolate was upregulated in the AO group.

**Figure 8:**
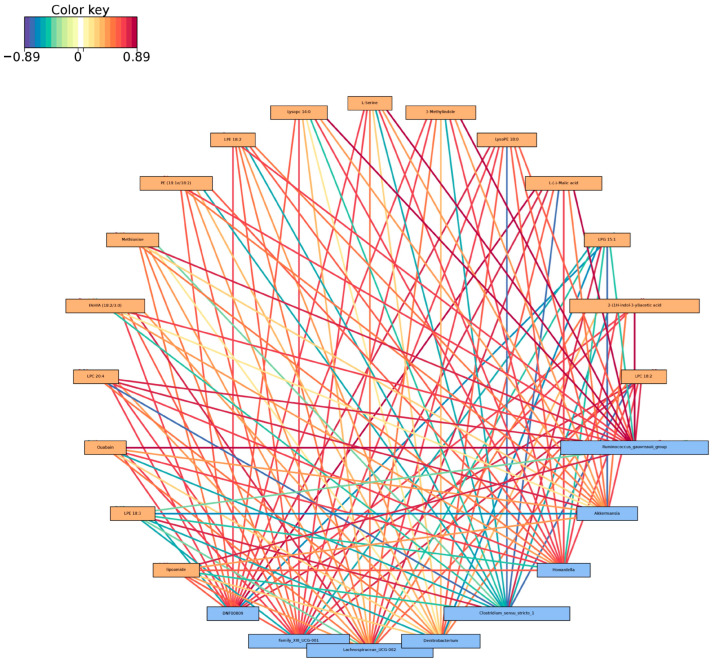
Heatmap of the correlation between the gut microbiota (genus) and metabolites of yak calves.

The association of differential metabolites with the top ten most abundant genera was examined using the Spearman statistical approach. The results revealed a significant negative association between Clostridium_sensu_stricto_1 and D-panthenol in the jejunum when comparing the AO group with the AH group (Figure 8). Notably, no associations were observed between metabolites and the remaining bacteria in the top ten at the genus level.

## 4. Discussion

Although calves are born with stomachs that are not fully developed to effectively utilize forage, they exhibit natural foraging behaviors at an early age [17]. While it is generally accepted that early intake of forage promotes rumen development and subsequent calf growth through physical stimulation, recent studies have shown that butyrate and propionate produced by carbohydrate-rich concentrates are more effective in promoting the development of the animals [18,19]. After weaning, calves fed on both concentrate and hay in the early stages had higher intake compared to calves fed only on concentrate or only on hay [20]. Therefore, feeding both concentrate and hay to lactating calves is more favorable for their growth and development.

According to this study, feeding calves mixed forage is better for their growth performance than feeding them separate forage. Yak calves in all three groups consumed the same quantity of dry matter, which was consistent with the results of previous studies indicating that diets with different roughage sources did not affect the dry matter intake of ruminants [21,22]. In this study, when half of the alfalfa hay was replaced with oat hay, the feed conversion rate of calves increased, resulting in the final weight of calves in the AO group being higher than that in the other two groups. Previous studies have found that the source of dietary NDF and the amount of NDF affect the growth performance of calves [23]. In this experiment, due to the complementary effect of nutrients between *Gramineae* and *Leguminosae*, the NDF content in the mixed forage we used was higher than that in the oat hay group or the alfalfa hay group. This can explain why the combined use of oat hay and alfalfa hay can enhance growth performance. This result is consistent with previous reports [24].

Animal health and productivity can be affected by changes in the variety of gut flora, as these changes are strongly linked to alterations in food absorption and immune system metabolism [25]. Previous studies have shown that the composition of the host’s diet and its own growth and development can also influence the diversity of bacteria in the gut [26]. Previous research has indicated that the supplementation of concentrates increases the level of nonstructural carbohydrates (NSCs), which stimulates the production of short-chain fatty acids (SCFAs) by intestinal bacteria, thus inhibiting the activity of acid-sensitive bacteria and decreasing microbial diversity [27]. In contrast, our experiment revealed an enhancement in the abundance and diversity of microorganisms within the jejunum and colon when oat hay was used as a substitute for alfalfa hay, despite the administration of the same concentrate. This observation is hypothesized to stem from the elevated neutral detergent fiber (NDF) content in oat hay, which beneficially impacts microbial diversity. Beta diversity revealed significant differences in the community composition of jejunum bacteria, especially when a combination of oat hay and alfalfa hay was used. PCA plots exhibited large variations among samples from the OH group, indicating that feeding oat hay alone had an unstable impact on the intestinal flora of yak calves.

Firmicutes, Proteobacteria, Bacteroidetes, and a small number of Actinomycetes are the main bacteria in the intestines of calves [12]. In this study, we observed that the dominant phyla were Firmicutes and Euryarchaeota in the jejunum and Firmicutes and Bacteroidetes in the colon. Firmicutes and Bacteroidetes are the dominant phyla in the gut of healthy mammals. The predominance of Firmicutes in the jejunum microflora of dairy and beef cattle has been shown to be associated with the high content of roughage in ruminant diets [28,29], and the proportion of Firmicutes in the gastrointestinal tract is significantly and positively correlated with the content of roughage in the diet [30]. Firmicutes contributes to the digestion of plant fiber and is mainly involved in the degradation of oligosaccharides, starch, and cellulose [31,32]. Firmicutes bacteria are rich in a large number of genes related to nutrient transporters, which promote the host’s absorption of carbohydrates and proteins [33]. Euryarchaeota, one of the main archaeal taxa found in the rumen of young ruminants, may colonize the ileum on the second day of life in newborn calves [34]. In this study, the dominant phylum in the jejunum was Euryarchaeota, and Methanobrevibacter was the dominant genus. The reason for this is that all calves were fed roughage, and the digestion products of roughage can provide the required carbon dioxide and hydrogen to Euryarchaeota. Methane is one of the end products of plant-based feed fermentation [35]. Bacteroidetes species are considered the most important in providing energy for yaks [36]. In this study, we found that at the phylum level, Bacteroidota and Spirochaetota abundance in the OH group was significantly higher than that in the AH group in the jejunum. Spirochaetota is pathogenic and exhibits resistance to the antibiotic ceftiofur sodium in newborn calves [37]. Previous studies have shown that flavonoids can reduce the content of Spirochaetota [38]. The AO group had significantly higher levels of Actinobacteria than the AH group. Actinobacteria has been reported to be an abundant phyla in newly weaned calves, and its abundance increases with an increase in dietary fiber intake [39]. This finding suggests that a diet consisting of oat hay mixed with alfalfa hay may be beneficial for future weaning.

At the genus level, *Romboutsia* and *Methanobrevibacter* dominated in the jejunum, whereas *Alistipes* and *UCG-005* dominated in the colon. *Romboutsia* is a butyric acid-producing bacterium that is more abundant in a healthy gut. It may play a key role in maintaining the health status of the host, and the absence of this microbial genus is a first indicator of an alteration in the mucosa [40]. *Methanobrevibacter* can produce methane from carbon dioxide, hydrogen, and formates. It can improve the function of cellulase activity. *UCG-005* is a genus of *Ruminococci*, which is related to the circulating level of acetic acid, which is also one of the most common volatile fatty acids produced by intestinal microbial fermentation [41]. It not only provides energy and nutrients for microbial growth and proliferation but also serves as a substrate for energy and biochemical reactions that can be utilized by the host [42,43]. This study further revealed that in the OH group, the abundances of UCG-005 and Alistipes increased significantly, whereas in the AO group, the abundance of Clostridium_sensu_stricto_1 decreased. According to a report by Xue [44], there is a connection between intestinal inflammatory illnesses and *Clostridium_sensu_stricto_1*. Additionally, the AO group had a considerably higher *Alistipes* abundance than the AH group in colon. Animal sickness and mental health are significant areas in which *Alistipes* is involved. The above results showed that replacing alfalfa hay with oat hay could increase the abundance of beneficial bacteria in jejunum and colon and could also decrease the abundance of harmful bacteria when replacing half of the oat hay with alfalfa hay. The structure and composition of the microbiome contribute to the maintenance of the intestinal environment’s stability, supporting the growing needs of various developmental stages and promoting a mature functional intestinal microbiome.

Different roughage ratios exerted significant effects on the concentration of intestinal metabolites. In the jejunum, both OH and AH groups exhibited the enrichment of differential metabolites, particularly serotonin, within specific KEGG pathways, including the “inflammatory mediator regulation of tryptophan (TRP) channels pathway”, “gap junction”, “synaptic vesicle cycle pathway”, and “taste transduction pathway”. Notably, serotonin levels were significantly downregulated in the OH group. Serotonin, a crucial product of tryptophan metabolism, plays a pivotal role in regulating intestinal peristalsis, with over 95% of its production distributed in the gastrointestinal tract [45]. This metabolite is predominantly found in EC cells of the intestinal mucosa and is implicated in inflammatory processes [46]. Our findings indicate that feeding on oat hay alone did not confer superior benefits in terms of jejunal peristalsis and immunity. In contrast, within the AO and AH groups, differential metabolites such as 3-methyl-2-oxobutanoic acid and panthenol exhibited significant enrichment in KEGG pathways related to “pantothenate and CoA biosynthesis” and “valine, leucine, and isoleucine degradation”. Additionally, D-panthenol was enriched in the “pantothenate and CoA biosynthesis” pathway, while L-palmitoylcarnitine was enriched in pathways linked to “fatty acid degradation” and “fatty acid metabolism”. Notably, all these differential metabolites were significantly upregulated in the AO group. It is noteworthy that 3-methyl-2-oxobutanoicacid serves as a precursor for pantothenic acid in Escherichia coli [47]. D-Panthenol exerts its effects on the gastrointestinal tract, enhancing lower intestinal motility. Furthermore, both 3-methyl-2-oxobutanoic acid and D-panthenol serve as precursors in the formation of coenzyme A, a crucial cofactor involved in various biochemical processes. Coenzyme A is integral to the breakdown of sugars, the oxidation of fatty acids, the catabolism of amino acids, the degradation of pyruvate, and the stimulation of the tricarboxylic acid cycle, which contribute significantly to meeting the body’s energy needs. Additionally, it supports the immune system in detoxifying harmful substances and activating white blood cells, thereby promoting hemoglobin synthesis and participating in antibody production. As vital acetyl-group and acyl-group carriers, CoA also facilitates the metabolism of fatty acids by catalyzing the breakdown and degradation of long-chain fatty acids through the addition or removal of acyl groups. It further assists in pyruvate oxidation and other acetylation reactions [48]. The effective metabolism of fatty acids, in turn, regulates the intestinal microbiota, fostering the growth of beneficial bacteria such as Lactobacillus and suppressing the proliferation of harmful bacteria, thereby enhancing intestinal microbial diversity [49]. 3-Methyl-2-oxobutanoic acid is also implicated in amino acid metabolism. According to studies, antibiotic-associated diarrhea is closely linked to the degradation of valine, leucine, and isoleucine [50]. Furthermore, methionine and L-serine were significantly enriched in various KEGG pathways, including “aminoacyl-tRNA biosynthesis”, “cysteine and methionine metabolism”, “protein digestion and absorption”, and “biosynthesis of amino acids”. Notably, L-serine was also enriched in the “glycine, serine, and threonine metabolism”, “sphingolipid metabolism”, and “sphingolipid signaling” pathways. The upregulation of amino acid metabolites observed in the AO group indicates that protein synthesis is facilitated by amino acid activation, a process catalyzed by aminoacyl-tRNA synthetases. Activated amino acids form aminoacyl-tRNAs with transfer RNAs (tRNAs), enabling the synthesis of peptide chains and proteins. This suggests that supplementary feeding, combined with regular feeding, may enhance microbial protein secretion and protein digestion and absorption. Additionally, certain proteins can regulate hormone levels, thereby maintaining homeostasis within the body’s internal environment [51]. The significant upregulation of these differential metabolites in the AO group indicates that the mixed feeding of alfalfa hay and oat hay is beneficial, promoting the production of favorable metabolites in the jejunum of yak calves.

In the OH and AH groups, differential metabolites such as adrenic acid, eicosapentaenoic acid, nervonic acid, and docosapentaenoic acid were enriched in the KEGG pathway related to the biosynthesis of unsaturated fatty acids. Additionally, metabolites such as N6,N6,N6-trimethyl-L-lysine, 5-hydroxy-L-lysine, L-hydroxylysine, and L-pipecolate were found to be enriched in the lysine degradation pathway. Notably, these differential metabolites exhibited significant downregulation in the OH group. Adrenic acid plays a crucial role in the regulation of inflammation and fatty acid metabolism in the body [52]. Eicosapentaenoic acid confers cardiovascular benefits by lowering triglyceride and non-HDL cholesterol levels [53]. Neuronic acid, a very long-chain monounsaturated fatty acid, is essential for the development of the brain’s nervous system and the treatment of neurological disorders [54]. Docosapentaenoic acid is primarily involved in the lowering of inflammation [55]. Collectively, these fatty acids act synergistically to modulate lipid metabolism in calves, indicating that the OH group may possess a reduced capacity for unsaturated fatty acid biosynthesis. Lysine, a crucial amino acid in pre-weaned calves, plays a pivotal role in enzyme and hormone synthesis, fat metabolism, and the enhancement of host immunity [56]. Furthermore, metabolites such as 4-hydroxybenzoic acid and 7,8-dihydrofolate were enriched in the KEGG pathway related to folate biosynthesis, while 3-methoxy-4-hydroxyphenylacetate was enriched in the dopaminergic synapse pathway. It is noteworthy that 4-hydroxybenzoic acid and 3-methoxy-4-hydroxyphenylacetate were downregulated, whereas 7,8-dihydrofolate was upregulated in the AO group. In both the OH group and the AH group, the enrichment of differentially expressed KEGG pathways and corresponding metabolites was identical. In contrast, the AO group exhibited a distinct pattern. 4-Hydroxybenzoate serves as an intermediate in numerous enzyme-mediated reactions in microorganisms. Folate is associated with cellulose degradation and plays a crucial role in the metabolism of animal DNA, RNA, and proteins. It also promotes the proliferation and differentiation of immune cells, as well as immunoglobulin production, thereby enhancing the disease resistance of animals. Additionally, folate maintains the normal function of the digestive system by influencing pancreatic secretion [57,58]. Dopamine, a member of the catecholamine family, is classically known for its role in regulating the central nervous system [59]. In summary, the combined feeding of oat hay and alfalfa hay to yak calves led to an increase in the concentration of beneficial metabolites.

The correlation analysis revealed that the decrease in *Clostridium_sensu _stricto_1* in the AO group compared to the AH group was associated with an increase in the metabolite D-panthenol. *Clostridium_sensu _stricto_1* is a genus of bacteria known to promote inflammatory responses, whereas D-panthenol is involved in the biosynthetic pathway of coenzyme A and pantothenic acid. The combination of oat hay and alfalfa hay was found to enhance the abundance of D-panthenol, thereby increasing the synthesis of pantothenic acid and coenzyme A. This pathway plays a crucial role in energy metabolism and fatty acid synthesis. It is hypothesized that the mixture of alfalfa and oat hay reduced the population of harmful bacteria and augmented energy metabolism and fatty acid synthesis, ultimately promoting calf growth and development.

## 5. Conclusions

In summary, it is feasible to replace alfalfa hay with oat hay in milk replacer and starter diets. Specifically, replacing half of the alfalfa hay with a combination of two forage types has been shown to increase the weight of yak calves, alter the diversity of intestinal microbiota, and facilitate the early establishment and improvement of intestinal microflora structure. This dietary modification results in significant changes in gut microbial metabolites, which exhibit positive associations with digestive functions, cofactor metabolism in the jejunum, and cofactor and vitamin metabolism in the colon. Overall, the combined use of these two forage types enhances nutrient absorption, boosts immune function, maintains internal homeostasis in yak calves, and ultimately benefits their growth and development.

## Figures and Tables

**Table 1:** Nutrient composition of the feed (% of dry matter).

Items	Milk Replacer	Alfalfa Hay	Oat Hay	Starter Feed
Dry matter (DM)	95.00	95.00	93.00	87.90
Crude protein (CP)	26.24	14.19	4.08	20.00
Ether extract (EE)	27.90	3.39	4.20	4.70
Neutral detergent fiber (NDF)	-	46.65	57.52	10.90
Acid detergent fiber (ADF)	-	37.50	31.51	4.10
Calcium (Ca)	2.50	1.55	1.05	0.80
Phosphorus (P)	1.40	0.74	0.15	0.45
Starch	-	2.34	2.72	34.35

**Table 2:** Effects of diet with oat hay as a substitute for alfalfa hay on growth performance and body size indexes of yak calves.

Items	Group			
	AH	OH	AO	*p*-Value
Initial body mass/kg	36.00 ± 1.58	35.80 ± 0.84	37.60 ± 0.55	0.053
Final body mass/kg	71.20 ± 1.79 ^b^	70.60 ± 0.55 ^b^	74.80 ± 3.11 ^a^	0.017
Average daily weight gain/(g/d)	293.33 ± 3.12	290.00 ± 3.12	310.00 ± 10.00	0.095
Feed-to-gain ratio	2.68 ± 0.03 ^a^	2.69 ± 0.01 ^a^	2.55 ± 0.048 ^b^	0.018
Dry matter intake/(kg/d)	1.00	1.00	1.00	1.000

^a,b^ Different superscript letters in the same row of data indicate significant differences (*p* < 0.05), while the absence of a letter indicates no significant difference (*p* > 0.05).

**Table 3:** Effect of oat hay as a substitute for alfalfa hay on OTUs and alpha diversity in the jejunum and colon of yak calves.

Items	Group			
	AH	OH	AO	*p*-Value
Jejunum				
OTUs	965.20 ± 89.90 ^b^	1058.80 ± 100.65 ^a,b^	1136.00 ± 44.75 ^a^	0.021
Chao1 index	966.75 ± 91.20 ^b^	1060.3 ± 101.15 ^a,b^	1138.97 ± 45.64 ^a^	0.021
Shannon index	8.33 ± 0.33	8.49 ± 0.28	8.73 ± 0.13	0.094
Simpson index	0.98 ± 0.005	0.99 ± 0.004	0.9933 ± 0.003	0.419
Colon				
OTUs	688.80 ± 78.92 ^b^	903.00 ± 106.30 ^a^	857.40 ± 120.65 ^a^	0.016
Chao1 index	690.68 ± 79.05 ^b^	905.45 ± 105.92 ^a^	860.33 ± 121.23 ^a^	0.016
Shannon index	6.73 ± 0.46 ^b^	7.47 ± 0.40 ^a^	7.49 ± 0.24 ^a^	0.012
Simpson index	0.97 ± 0.02	0.98 ± 0.006	0.97 ± 0.01	0.225

^a,b^ Different superscript letters in the same row of data indicate significant differences (*p* < 0.05), while the absence of a letter indicates no significant difference (*p* > 0.05)

**Table 4:** KEGG pathways enriched in jejunal differential metabolites and the corresponding differential metabolites for each pathway.

Items	Map Title	*p*-Value	Metabolites
Positive ion mode			
AH vs. OH	Inflammatory mediator regulation of TRP channels	0.012	Serotonin↑
	Gap junction	0.036	
	Synaptic vesicle cycle	0.036	
	Taste transduction	0.047	
AH vs. AO	Pantothenate and CoA biosynthesis	0.027	3-Methyl-2-oxobutanoic acid↓ D-Panthenol↓
	Fatty acid degradation	0.048	L-Palmitoylcarnitine↓
	Valine, leucine, and isoleucine degradation	0.048	3-Methyl-2-oxobutanoic acid↓
	Fatty acid metabolism	0.048	L-Palmitoylcarnitine↓
Negative ion mode			
AH vs. AO	Aminoacyl-tRNA biosynthesis	<0.01	Methionine↓ L-Serine↓
	Cysteine and methionine metabolism	<0.01	
	Protein digestion and absorption	<0.01	
	Biosynthesis of amino acids	0.020	
	Glycine, serine, and threonine metabolism	0.030	L-Serine↓
	Sphingolipid metabolism	0.030	
	Sphingolipid signaling pathway	0.030	

“↑” means upregulated metabolite; “↓” means downregulated metabolite.

**Table 5:** KEGG pathways enriched in colon differential metabolites and the corresponding differential metabolites for each pathway.

Items	Map Title	*p*-Value	Metabolites
Positive ion mode			
AH vs. OH	Biosynthesis of unsaturated fatty acids	<0.01	Adrenic acid↑ Eicosapentaenoic acid↑ Nervonic acid↑ Docosapentaenoic acid↑
	Lysine degradation	<0.01	N6,N6,N6-Trimethyl-l-lysine↑ 5-Hydroxy-l-lysine↑ l-Hydroxylysine↑ l-Pipecolate↑
Negative ion mode			
AH vs. OH	Folate biosynthesis	0.020	4-Hydroxybenzoic acid↑ 7,8-Dihydrofolate↓
	Dopaminergic synapse	0.020	3-Methoxy-4-hydroxyphenylacetate↑
AH vs. AO	Folate biosynthesis	<0.01	4-Hydroxybenzoic acid↑ 7,8-Dihydrofolate↓
	Dopaminergic synapse	<0.01	3-Methoxy-4-hydroxyphenylacetate↑

“↑” means upregulated metabolite; “↓” means downregulated metabolite.

## Data Availability

The original contributions presented in the study are included in the article; further inquiries can be directed to the corresponding authors.
